# Taking a corneal scrape and making a diagnosis

**Published:** 2009-12

**Authors:** Astrid Leck

**Affiliations:** Research fellow, International Centre for Eye Health, London School of Hygiene and Tropical Medicine, Keppel Street, London W1CE 7HT, UK.

**Figure:**
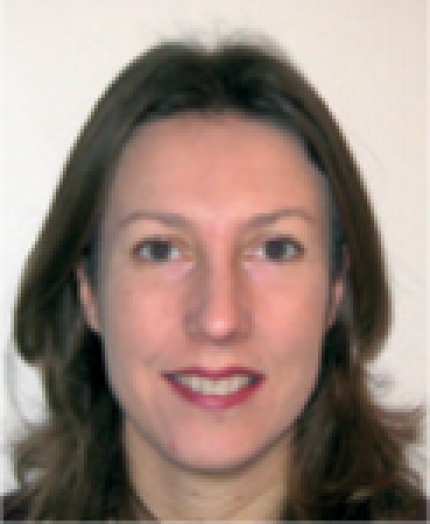


This article aims to provide a comprehensive guide to taking a corneal scrape and making a diagnosis. However, there are settings in which there are either limited or no laboratory facilities available to the ophthalmologist; for example, at primary level eye care centres in rural locations. In these circumstances, microscopy may still provide valuable information to guide the clinician in their choice of treatment (Figures 5–11 are images of infected corneal tissue as seen by microscopy).

**Figure:**
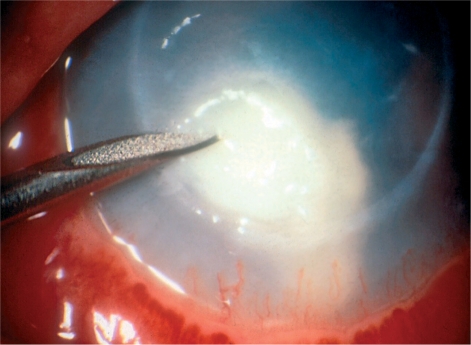
Figure 1. Taking a corneal scrape

J Dart

## Taking a corneal scrape

What you will need:

- **21-guage needles or Kimura scalpel**
- **2x clean microscope slides**
- **1x blood agar plate (FBA)**
- **1x Sabouraud glucose agar plate (SGA)**
- **1x brain heart infusion broth (BHI) (for fastidious organisms)**
- 1x cooked meat broth (CMB) (excludes facultative anaerobes)
- 1x thioglycollate broth (TB)
- 1x non-nutrient agar (NNA) (if *Acanthamoeba sp.* is suspected)

In order to have the best possible chance of providing the clinician with an accurate diagnosis, all the media listed are required. In some remote settings, some media may not be available or there may be limitations in the variety of media it is possible to process. For these situations, the minimum requirements are denoted above in **bold type**, in order of importance. Liquid phase media (broths) must be used when available. If only one liquid phase media is to be used, this should be BHI; it is essential to inoculate more than one bottle. NNA is indicated only if amoebic infection is suspected.

### General principles

- If possible, withdraw the use of antimicrobial agents for 24 hours prior to sampling. Where this is not possible, the use of liquid phase media, for example BHI, serves as a diluent that reduces the concentration of the drug below the minimum inhibitory concentration (MIC).
- Apply anaesthetic drops that do not contain preservative.
- Use a different needle to take each specimen or, if using a Kimura scalpel, flame the scalpel between samples.
- If fungal or amoebic infection is suspected, it is preferable to sample material from the deeper stromal layer of the cornea.

Order of specimen preparation:

1. Slide for Gram stain and slide for alternative staining processes
2. Solid phase media (FBA/HBA, SGA, NNA)
3. Liquid phase media (BHI, CMB, TB)

If the ulcer is very discrete or only a small amount of corneal material is available, inoculate one solid and one liquid phase medium.

### Specimen collection for microscopy

- Label slide with patient's name, date of birth, and hospital number.
- Draw/etch a circle on the slide and place specimen within the circle (Figure 2).
- Air-dry and cover with a protective slide (tape the ends) or place in a slide transport box.

**Figure:**
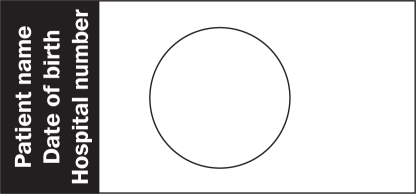
Figure 2. Slide with label and circle for placing the specimen

### Inoculating culture media

- Gently smear material on the surface of agar in C-streaks (Figure 3); taking care not to puncture the surface of the agar.
- Sellotape the lid of the plate to the base around the perimeter.
- Incubate inoculated culture media as soon as possible. Refrigeration of specimens is to be discouraged and, if not being transported directly to the laboratory, it is preferable to keep samples at room temperature.

**Figure:**
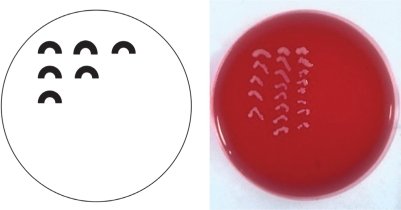
Figure 3. Smear the material on the surface of agar in C-streaks

Astrid Leck

## Making a diagnosis

### Microscopy: the Gram stain

1. Air-dry and heat-fix specimen using a Bunsen burner or spirit lamp
2. Allow slide to cool on staining rack
3. Flood slide with crystal violet; leave for 1 minute (Figure 4)
4. Rinse slide in clean running water
5. Flood slide with Gram's iodine; leave for 1 minute
6. Rinse slide in clean running water
7. Apply acetone and rinse immediately under running water (exposure to acetone <2 seconds)
8. Counter-stain with carbol fuschin for 30 seconds
9. Rinse in clean running water then dry with blotting paper
10. View specimen with 10x objective
11. Place a drop of immersion oil on the slide and view with 100x oil-immersion objective.

**Figure:**
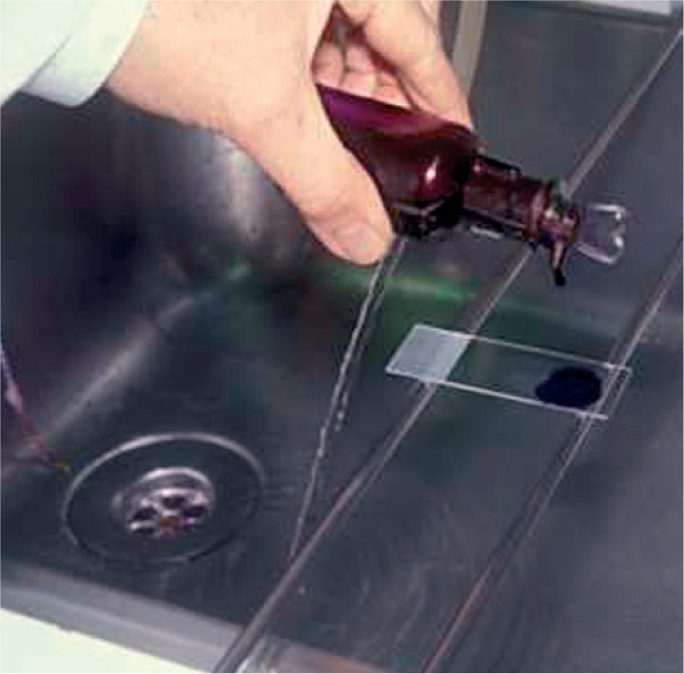
Figure 4. Flood the slide with crystal violet

Astrid Leck

- Gram positive (+ve) cocci most commonly associated with suppurative keratitis are the *Staphylococci* (Figure 5) and *Streptococci* (Figure 6, *Streptococcus pneumoniae*).
- Gram negative (−ve) bacilli, such as *Pseudomonas sp.* (Figure 7), may be associated with corneal infection.
- A definitive diagnosis of *Nocardia sp* (Gram variable) may be possible
- Yeast cells will stain positively.

**Figure:**
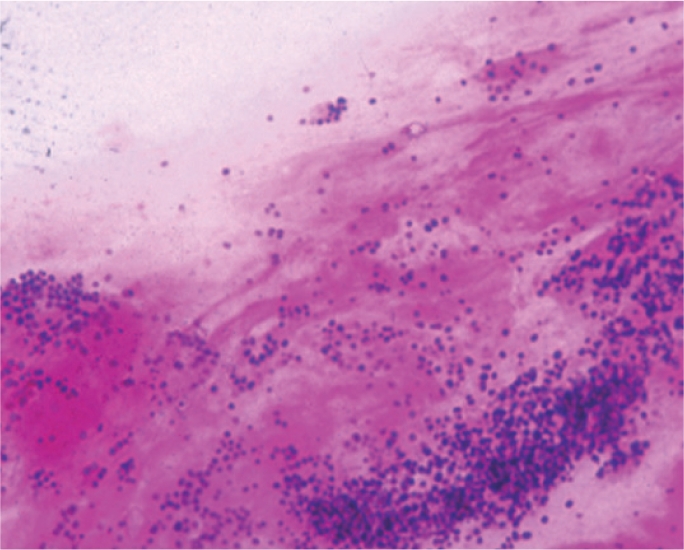
Figure 5. Staphylococci sp.

MM Matheson

**Figure:**
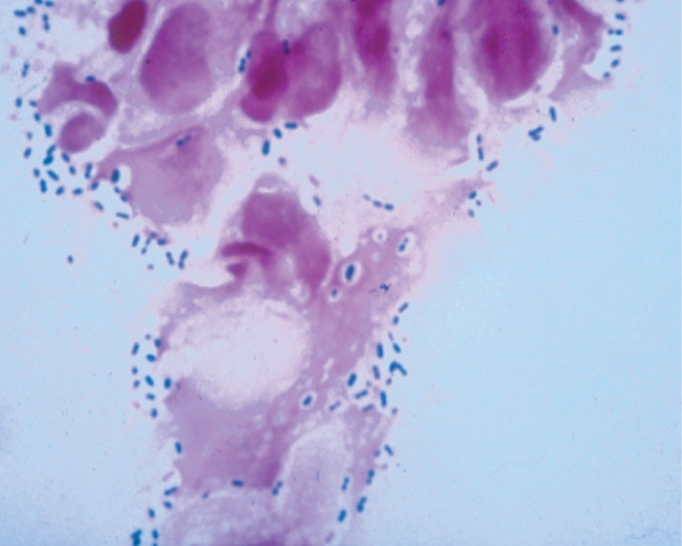
Figure 6. Streptococcus pneumoniae

MM Matheson

**Figure:**
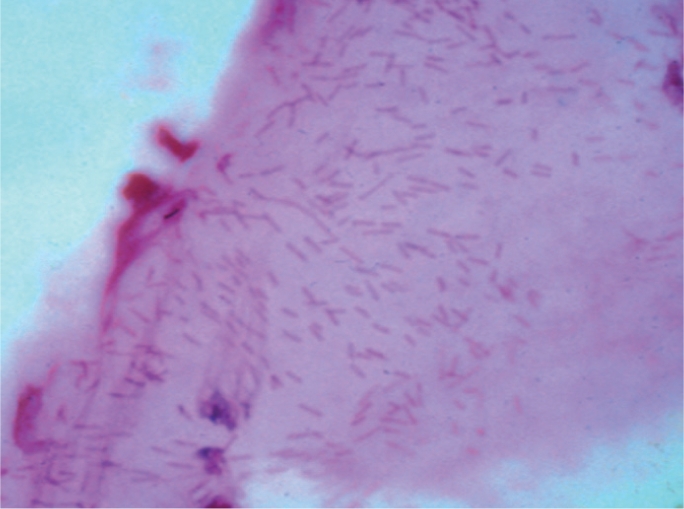
Figure 7. Pseudomonas sp.

MM Matheson

Although not the first choice of stains for fungi, yeast cells, pseudohyphae, and fungal hyphae may be visualised in Gram-stained corneal material, typically staining negatively or Gram variable. For microscopy to provide a more definitive diagnostic tool for fungal infection, Gram stain can be destained and restained using a more appropriate stain (Figures 8 and 9).

**Figure:**
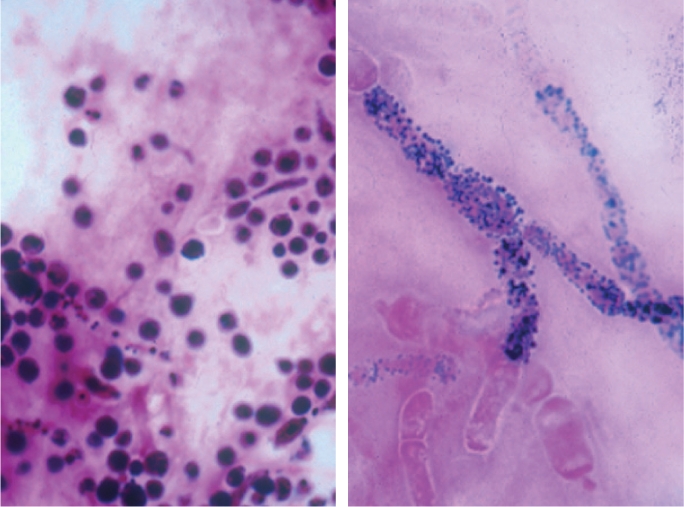
Figure 8. Gram appearance of yeast cells (left) and pseudohyphae (right)

Astrid Leck

**Figure:**
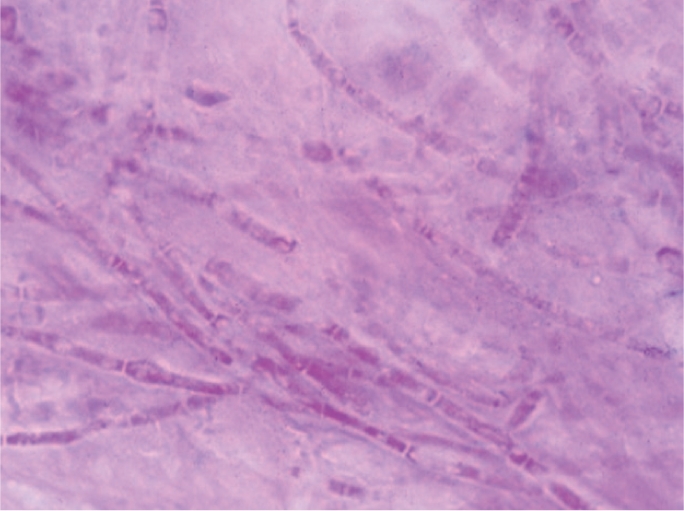
Figure 9. Fungal hyphae visible after Gram stain

Astrid Leck

### Microscopy: additional methods

Lactophenol cotton blue (LPCB) or potassium hydroxide (KOH) wet mount preparations are used to visualise fungi (Figure 10).

**Figure:**
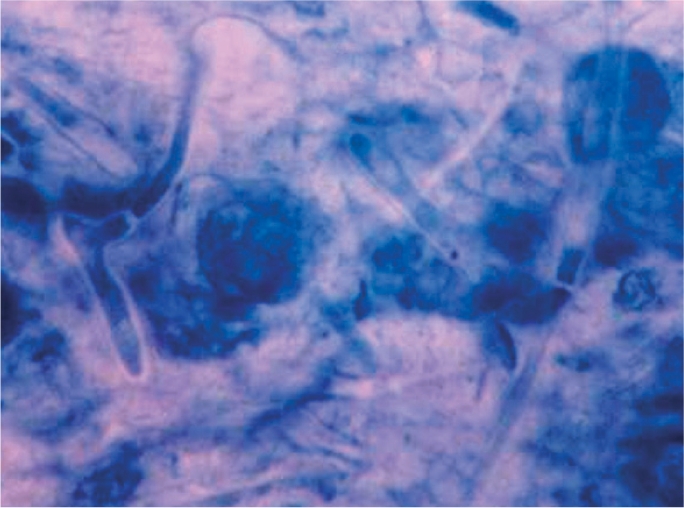
Figure 10. Fungal hyphae stained with lactophenol cotton blue

PA Thomas

1. Add a drop of lactophenol cotton blue mountant to the slide.
2. Holding the coverslip between your forefinger and thumb, touch one edge of the drop of mountant with the coverslip edge, the lower it gently, avoiding air bubbles. The preparation is now ready.
3. Initial observation should be made using the low power objective (10x), switching to the higher power (40x) objective for a more detailed examination.
4. Calcofluor white and Periodic Acid Schiff reaction (PAS) staining may also be used.

## Diagnostic criteria

### Diagnostic criteria applied to bacterial culture

- the same organism growing at the site of inoculation on two or more solid phase cultures, or
- growth at site of inoculation on one solid phase media of an organism consistent with microscopy, or
- confluent growth on one media.

### Diagnostic criteria applied to fungal specimens

- fungal hyphae observed in corneal specimen stained on microscopic examination, or
- growth at site of inoculation on solid culture media

## Amoebic infections

The cyst form of *Acanthamoeba sp.* can be visualised in corneal material using a direct fluorescent technique such as calcofluor white (Figure 11), haemotoxylin and eosin, LPCB, or PAS. If corneal infection with *Acanthamoeba sp.* is suspected, inoculate corneal material onto non-nutrient agar in a demarcated area of the plate. In the laboratory, the square of agar where the specimen was inoculated will be excised and inverted onto an NNA plate seeded with a lawn of *E. coli.* Growth of the trophozoite form is imperative to confirm viability of the organism and thus prove it to be the organism responsible for infection (Figure 12).

**Figure:**
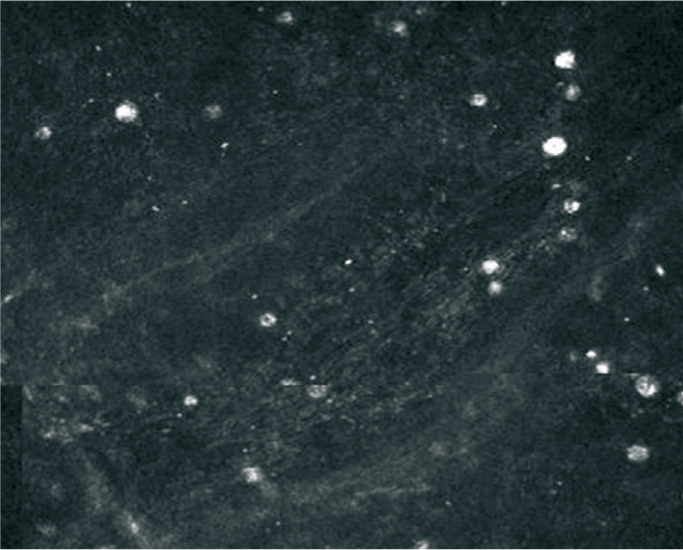
Figure 11. Calcofluor white preparation

J Dart

**Figure:**
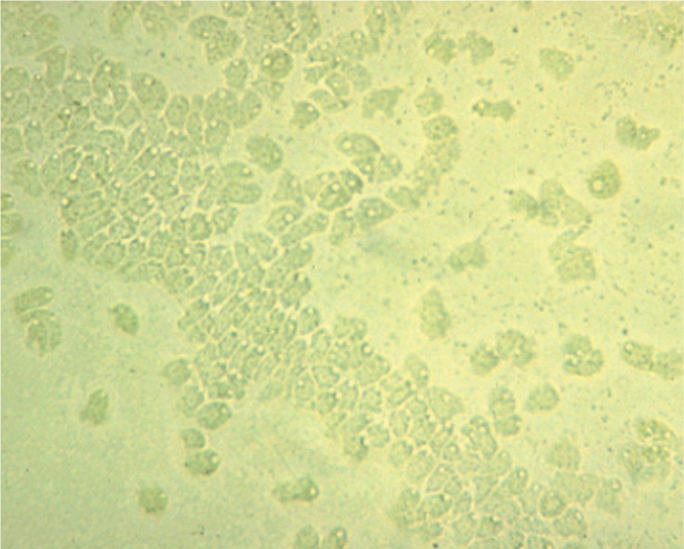
Figure 12. The trophozoite form of Acanthamoeba

Astrid Leck

